# AI-Enabled Personalized Smoking Cessation Intervention With the Aipaca Chatbot: Mixed Methods Feasibility Study

**DOI:** 10.2196/73319

**Published:** 2025-12-11

**Authors:** Yunlong Liu, Paul Calle, Mariah Vadakekut, Daniel Rubin, Zsolt Nagykaldi, Mark Doescher, Lisa Hightow-Weidman, Chongle Pan, Ruosi Shao

**Affiliations:** 1 School of Computer Science University of Oklahoma Norman, OK United States; 2 Department of Community Health and Family Medicine College of Medicine University of Florida Gainesville, FL United States; 3 Department of Family and Preventive Medicine College of Medicine University of Oklahoma Health Sciences Center Oklahoma City, OK United States; 4 College of Nursing Florida State University Tallahassee, FL United States; 5 College of Communication and Information Florida State University Tallahassee, FL United States

**Keywords:** generative AI, conversational agent, usability, tobacco treatment, health intervention

## Abstract

**Background:**

Tobacco use remains the leading cause of preventable mortality in the United States; yet, evidence-based cessation services remain underused due to staffing constraints, limited access to counseling, and competing clinical priorities. Generative artificial intelligence (GenAI) chatbots may address these barriers by delivering personalized, guideline-aligned counseling through naturalistic dialogue. However, little is known about how GenAI chatbots support smoking cessation at both outcome and communication process levels.

**Objective:**

This feasibility study evaluated the implementation of an evidence-based smoking cessation counseling session delivered by a GenAI-powered chatbot, Aipaca. We examined (1) pre-post changes in cessation preparedness, (2) communication dynamics during counseling sessions, and (3) user perceptions of the chatbot’s value, limitations, and design needs.

**Methods:**

We conducted an observational, single-arm, mixed methods study with 29 adult smokers. Participants completed pre-post surveys measuring knowledge of smoking-related health risks and cessation methods, self-efficacy, and readiness to quit. Each engaged in a 30-minute text-based counseling session with Aipaca, powered by GPT-4 and structured using the 5A’s framework (Ask, Advise, Assess, Assist, Arrange). Sessions were transcribed for microsequential conversation analysis. Twenty-five participants completed semistructured interviews exploring perceived value, challenges, and design suggestions. Quantitative data were analyzed with paired-samples *t* tests, qualitative data were thematically analyzed, and transcripts were analyzed for interactional practices. The methodological strength of this study lies in its triangulated approach, which combines quantitative measurement of intervention effectiveness, qualitative analysis of user interviews, and conversational analysis of counseling transcripts to generate a comprehensive understanding of both outcomes and underlying mechanisms.

**Results:**

Participants demonstrated significant improvements in all preparedness indicators: knowledge of health risks, knowledge of cessation methods, self-efficacy, and readiness to quit. Conversation analysis identified three recurrent patterns enabling counseling-relevant dynamics: (1) contextual referencing and continuity, (2) formulations with elaboration prompts, and (3) narrative progression toward collaborative planning. Interview themes underscored Aipaca’s perceived value as an accessible, nonjudgmental, and motivating resource, capable of delivering personalized and interactive support. Criticisms included limited accountability, reduced cultural resonance, and overly goal-directed style. Participants emphasized design needs such as proactive engagement, gamified progress tracking, empathetic or anthropomorphic personas, and safeguards for accuracy.

**Conclusions:**

This mixed methods feasibility study demonstrates that GenAI can deliver evidence-based smoking cessation counseling with measurable short-term gains in cessation preparedness and process-level communication patterns consistent with motivational interviewing. Users valued Aipaca’s accessibility, empathy, and personalization, while also articulating expectations for richer social roles and long-term accountability. Findings highlight both the promise and challenges of integrating GenAI into digital health: pairing adaptive language generation with human-centered design, embedding accuracy safeguards, and ensuring integration into multilevel cessation infrastructures will be essential for future clinical deployment.

## Introduction

### Background

Tobacco use remains the leading cause of preventable mortality in the United States, accounting for more than 480,000 deaths each year, nearly 1 in 5 of all US deaths [1]. Smoking drives premature death through its role in diseases such as lung cancer, chronic obstructive pulmonary disease, and coronary artery disease [2]. Implementing evidence-based tobacco treatment interventions is therefore a continuing public health imperative [3]. While these in-person interventions are effective in both the short and long term, their widespread implementation is often hindered by high demands on staff and clinical resources, limited access to in-person counseling, and competing priorities for clinicians’ time [4-6]. These persistent barriers highlight the need for scalable, innovative approaches to complement existing cessation services.

Recent advancements in generative artificial intelligence (GenAI) have created new opportunities for implementing evidence-based health interventions through conversational agents [7-10]. GenAI-powered chatbots are uniquely positioned to deliver highly personalized, interactive, and natural language-based counseling, adapting in real time to users’ evolving needs and preferences [11]. This potential for dynamic, human-like dialogue has not only expanded the technical capabilities of chatbots but also raised users’ expectations regarding naturalness, engagement, and the fulfillment of complex social roles in digital health.

Despite these advances, the mechanisms by which GenAI-driven chatbots influence health behavior change, particularly through communication processes and user perceptions, remain poorly understood. Most published studies have focused either on quantitative outcomes or on qualitative user interviews but seldom combine these with in-depth analysis of the counseling conversations themselves. Methodological triangulation, combining quantitative measures of intervention outcomes, qualitative interviews of user perceptions, and conversational analysis of the counseling process and communication dynamics between participants and artificial intelligence (AI) chatbots, strengthens research by enabling a more comprehensive understanding of complex phenomena [12]. In the context of digital health, triangulation that encompasses quantitative effectiveness, qualitative interviews, and conversational analysis of transcripts is particularly valuable for elucidating both what works and how it works.

### Related Work

#### The Rise of Conversational Agents and GenAI in Substance Use Intervention

Conversational agents (CAs), such as chatbots and avatar-led systems, have increasingly been adopted to deliver tobacco treatment and broader substance use interventions. A systematic review of 13 studies (N=8236) found that CAs significantly improved cessation outcomes (sample-weighted odds ratio 1.66, 95% CI 1.33-2.07) and were generally well-accepted by users, commonly assessed via self-reported satisfaction, perceived ease of use, comfort interacting with the agent, and willingness to recommend [13]. Chatbot-delivered interventions vary from 4 to 24 weeks, with interaction frequencies ranging from daily to multiple times per week, and median engagement levels around 36 of 42 intervention days. Engagement was high, with over 85% of participants initiating at least one interaction and about 75% reading all messages [13]. Similarly, a meta-analysis of 5 randomized controlled trials (RCTs; N=58,796) revealed that conversational AI interventions significantly increased 6-month abstinence rates compared to control conditions (risk ratio [RR] 1.29, 95% CI 1.13-1.46) [14]. These trials examined CA in various delivery formats, such as chatbots embedded in smoking cessation apps with daily interactive counseling, social media–based CAs embedded in group chats, and internet-based avatars delivering acceptance and commitment therapy (ACT) [15-17]. Control groups across these RCTs included usual care with in-person counseling and pharmacotherapy, smoking-cessation apps without chatbot functionality, or static smoking cessation tips with no interactive support.

Evidence from single-arm and pilot trials extends these findings to specific populations, including young adults and sexual and gender minority users. For example, avatar-led ACT interventions improved self-efficacy and quit intentions among university students through six 25-minute digital sessions, with 51.9% of completers achieving abstinence at postintervention [18]. Similarly, a digital ACT-based program tailored for sexual and gender minority young adults yielded 34% biochemically confirmed abstinence at postintervention, approximately 3 times higher than the 9%-12% rates observed in previous digital interventions [19]. Beyond cessation counseling, CAs have also been applied to screening and intake. Jeanne, a 3D embodied CA designed to assess tobacco and alcohol use disorders, can conduct a 4.4-minute structured interview using validated measures (eg, Cigarette Dependence Scale-5 for tobacco) [20]. Findings indicated high acceptability (mean score=24.8/30), strong concordance with paper-based tools (*r*>0.89), and excellent diagnostic validity (eg, area under the curve=0.97 for tobacco), supporting the feasibility of automating early stages of behavioral health interventions.

Taken together, existing evidence supports the initial feasibility and effectiveness of CAs across the tobacco treatment continuum, including screening, initial counseling, quit planning, motivational interviewing (MI), relapse prevention, and follow-up coaching. However, barriers were identified, especially for earlier rule-based or prescripted systems, including a lack of human-likeness, limited response coherence, and constrained conversational depth, which hindered relational communication, reduced engagement, and compromised long-term intervention effectiveness [13]. Moreover, attrition rates remain substantial in CA-based trials, ranging from 14% to 80% across studies, reflecting heterogeneous study durations and definition of “dropout,” and underscoring engagement as a persistent challenge [14]. Importantly, this engagement challenge is broadly comparable to other cessation modalities: for example, 50% of participants dropped out during treatment, and 61% were not reached at 7-month follow-up in a large state Quitline cohort (N=49,347) [21].

#### Chatbot Design and Integration of Evidence-Based Intervention

Chatbot-delivered smoking cessation interventions increasingly combine structured, evidence-based counseling components with human-centered design principles and, in many cases, generative AI capabilities. Systematic reviews indicate that effective systems typically integrate multiple intervention elements through frequent, tailored, multiweek interactions, encompassing screening and intake, initial counseling and psychoeducation, MI, personalized quit-plan development, skill-building and coping strategies, relapse prevention, and referral to human services [13,14]. For example, Florence, the World Health Organization’s web-based virtual human, provides 24/7 access to guideline-aligned brief tobacco treatment, assisting users in setting quit dates, developing quit plans, and connecting to local quitlines and cessation resources [22]. Evidence-based MI and cognitive behavioral therapy (CBT) approaches have also been integrated into chatbot systems. A web-based MI-style chatbot delivers 2 structured MI sessions: an assessment session of smoking history and quitting intentions, followed by a personalized normative feedback session designed to elicit reasons for quitting [23]. Roby, another chatbot-based intervention, provides a 5-session program that combines MI and CBT, guiding users through quit date setting, withdrawal and craving management, and relapse prevention [24]. Despite these advances, substantial variability remains in the reporting of conversational design and fidelity, particularly in how chatbots structure sessions and implement features such as content sequencing, turn-taking, prompt logic, and follow-up cadence [25].

Across development efforts, human-centered design and iterative co-design processes have been central to optimizing usability, therapeutic relevance, and long-term engagement of smoking cessation chatbots. The development of QuitBot followed a 4-year, 11-step user-centered design process that included needs assessments, iterative prototyping, diary studies, and a pilot RCT; user feedback led to refinements in persona, tone, session flow, and eventually the addition of an open-ended Q&A function supported by fine-tuned large language models (LLMs) [25]. Similar iterative design principles guided the World Health Organization’s Florence chatbot, which was codeveloped with user testing to ensure accessibility, personalization, and integration with local cessation resources [22]. Systematic reviews reveal that users consistently value design elements that enhance empathy, interactivity, and personalization, while expressing dissatisfaction with rigid, scripted conversations or excessive repetition [13,14]. Evidence from human-centered studies further highlights that users prefer supportive personas, clear quit-planning tools, flexible frequency of interaction, and integration of progress tracking features [25].

The natural language processing (NLP) techniques underpinning smoking cessation chatbots have evolved from prescripted and retrieval-based systems to more advanced generative AI models. Early systems, such as Florence, relied on prescripted content to deliver guideline-based brief interventions with high safety and fidelity, but limited conversational depth and flexibility [22]. Retrieval-based designs introduced greater adaptability by using intent classification and predefined response libraries, as seen in TAMI, the tobacco addiction management intelligent agent [26]. TAMI incorporated a human-in-the-loop approach, in which uncovered intents and user inputs were iteratively analyzed and mapped to new response templates, thereby extending the flexibility and coverage of its conversations while maintaining control and consistency. More recent systems leverage generative models to create dynamic, context-aware counseling exchanges. For example, MIBot used a hybrid design that combined scripted prompts with fine-tuned open-source transformer models to generate MI-style reflections [27]. Subsequent evaluations showed that user satisfaction with MIBot decreased after the release of ChatGPT, reflecting users’ rising expectations for conversational empathy and coherence. In addition, users with prior ChatGPT experience produced longer responses and reported greater increases in the perceived importance of quitting smoking, suggesting that exposure to generative AI systems is shaping user expectations of therapeutic chatbots in meaningful ways. Beyond LLMs, reinforcement learning has also been applied to optimize persuasive message strategies by modeling user traits and motivational states [28]. To date, most research-oriented cessation chatbots use open-source fine-tuned models (eg, GPT-2/3 derivatives) or custom NLP pipelines, whereas clinical deployment of closed-source commercial models such as OpenAI’s GPT-4/5 remains limited, reflecting both the technical opportunities and ongoing concerns regarding safety, bias, and transparency.

### Study Objectives

This feasibility study used a mixed methods design to evaluate the implementation of an evidence-based smoking cessation intervention delivered by a GenAI-powered chatbot. We recruited 29 adult smokers to participate in an initial counseling session with the AI chatbot, Aipaca (AI for Patient Care), during which each participant developed a personalized quit plan and set a quit date in accordance with the U.S. Public Health Service guideline [29]. Following this session, participants completed a structured interview to share their experiences, perceptions, and suggestions for improvement. We further conducted conversational analysis with all counseling transcripts to examine communication dynamics between users and the AI.

The methodological strength of this study lies in its triangulated approach, which combines quantitative measurement of intervention effectiveness, qualitative analysis of user interviews, and conversational analysis of counseling transcripts to generate a comprehensive understanding of both outcomes and underlying mechanisms [12]. Our study addressed three key questions: (1) How effectively can a GenAI chatbot deliver smoking cessation counseling? (2) What communication dynamics arise during user interactions with the chatbot? (3) How do users perceive the chatbot’s role in their cessation efforts?

By integrating these perspectives, our work contributes to the growing literature on AI-augmented health interventions and provides practical guidance for the human-centered design and evaluation of GenAI-powered chatbots in evidence-based behavioral counseling.

## Methods

### Chatbot Development

#### System Architecture and Implementation

**Figure 1:**
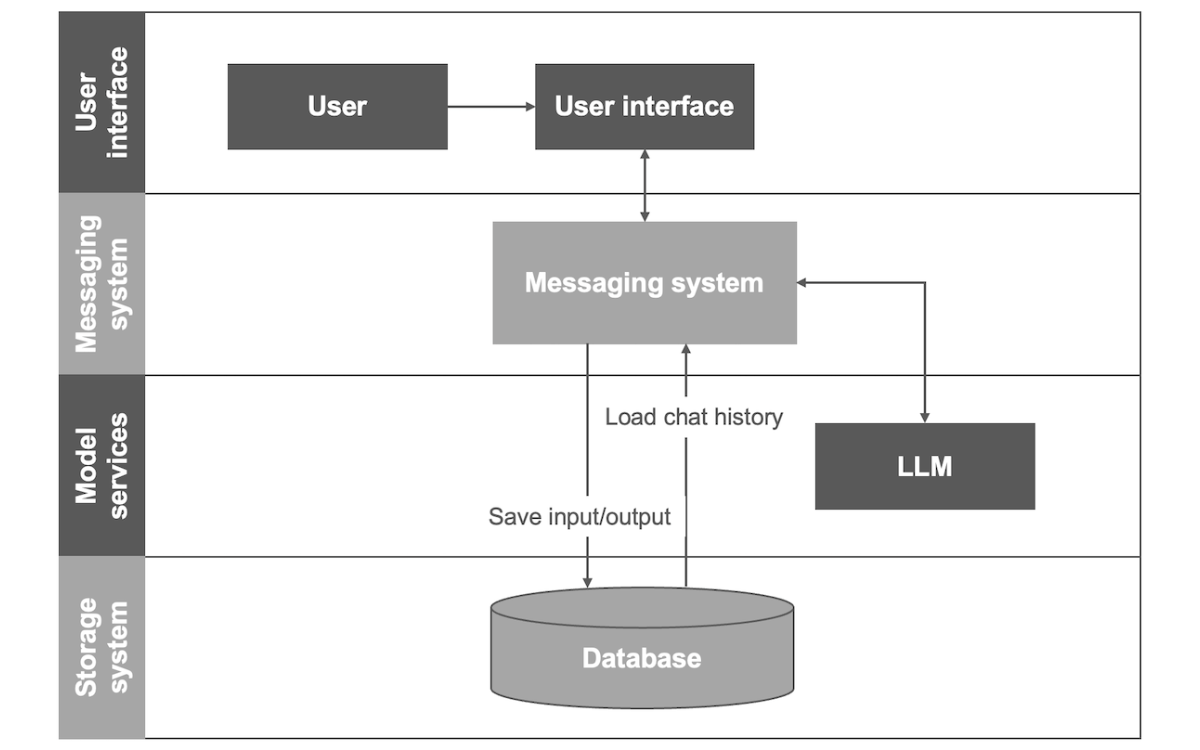
The system architecture of the Alpaca chatbot consists of four key components: user interface (web front end used by participants), messaging system (session management, routing, logging, and attention-check handling), model services (LLM for counseling responses), and storage system (database saving deidentified inputs/outputs and loading chat history). Arrows indicate data flow during the counseling sessions. LLM: large language model.

The text-based chatbot, Aipaca, was developed using the GPT-4 API [30], which enables interactive and personalized responses through the generative pretrained transformer model. The system architecture consists of 4 key components: user interface, messaging system, model service, and storage system (Figure 1). The front-end user interface was designed using HTML and JavaScript, accessible via web browsers and smartphones. It incorporated design enhancements such as auto-scrolling during conversations and a pop-up displaying a survey link upon completion of the counseling session. The messaging system manages communication between users and model services, facilitating user authentication through Google sign-in or a 6-digit user ID, implemented with Python and RESTful APIs. It handles request routing and chat history management, with key endpoints for login, menu retrieval, chat initiation, response generation, survey access, and chat history retrieval. JSON is used for data interchange, ensuring efficient and secure communication across the system. Each model service operates independently, using Python Flask to enable a modular and scalable architecture. This setup allows for seamless integration of additional APIs, advanced models, or increased server capacity to support future enhancements. Independent operation across different machines or ports further enhances system flexibility and scalability. The storage system offers 2 methods for information retrieval: users authenticated through Google sign-in can access their complete chat history, while those using a 6-digit ID can retrieve chat history from the past 2 hours, accommodating the typical duration of an initial counseling session (approximately 30 minutes). This setup is optimized for initial counseling interactions and aligns with the cross-sectional nature of our study design, ensuring secure authentication and efficient data management.

#### Implementing Evidence-Based Smoking Cessation Counseling Through Prompting

**Figure 2:**
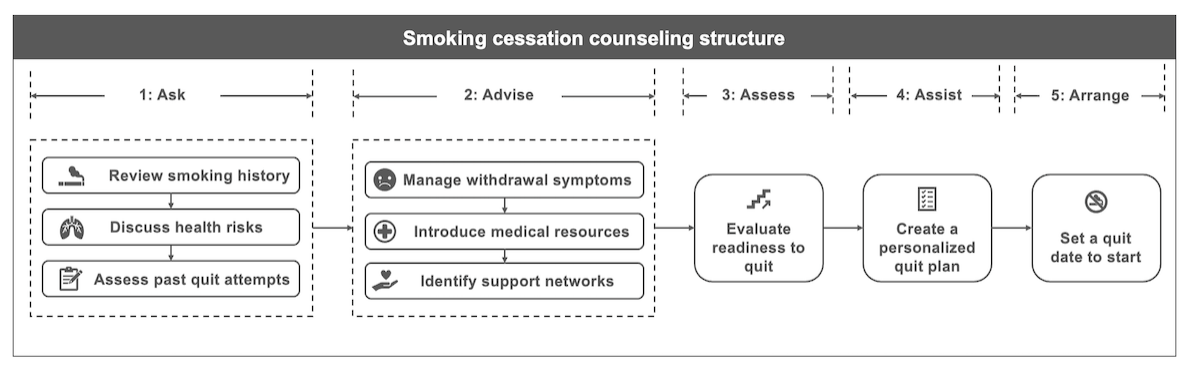
Five-step counseling structure following the 5A framework. Intervention workflow for a guideline-concordant, evidence-based session (approx. 10-15 minutes) delivered to adult US smokers in a single-arm study. Steps include ask (review smoking history, discuss health risks, and assess past quit attempts), advise (manage withdrawal, introduce evidence-based resources, and identify support), assess (evaluate readiness to quit), assist (create a personalized quit plan), and arrange (set a quit date).

The evidence-based standards of practice developed by the Tobacco Treatment Specialist (TTS) training program were implemented in Aipaca through structured prompting [31]. The prompt outlined Aipaca’s role as a TTS and provided a comprehensive framework for conducting initial cessation counseling. This framework emphasized a client-centered, MI approach, encouraging participants to openly discuss their tobacco use, reflect on smoking triggers, identify strategies for behavior change, and develop coping mechanisms to support cessation. The counseling structure adhered to the 5A’s framework (Ask, Advise, Assess, Assist, Arrange) as outlined in clinical practice guidelines [4,32], and was operationalized into a five-step process (Figure 2): (1) asking about tobacco use and previous quit attempts, (2) advising on tailored quitting strategies and resources, (3) assessing readiness to quit, (4) assisting in developing a personalized quit plan, and (5) arranging a quit date.

The prompting framework underwent iterative pilot testing and refinement by 3 certified TTS through simulated counseling sessions, incorporating insights from clinical practice and case-based scenarios in tobacco treatment. Consistent with clinical practice guidelines, the initial counseling session was designed to last 10-15 minutes, providing low-to-medium intensity counseling [4]. Prior to study launch, we evaluated Aipaca’s performance using a standard TTS certification exam, a requirement for accredited TTS training programs by the Council for Tobacco Treatment Training Programs. Aipaca achieved a score of 81%, exceeding the required passing threshold of 70%.

### Study Design

**Figure 3:**
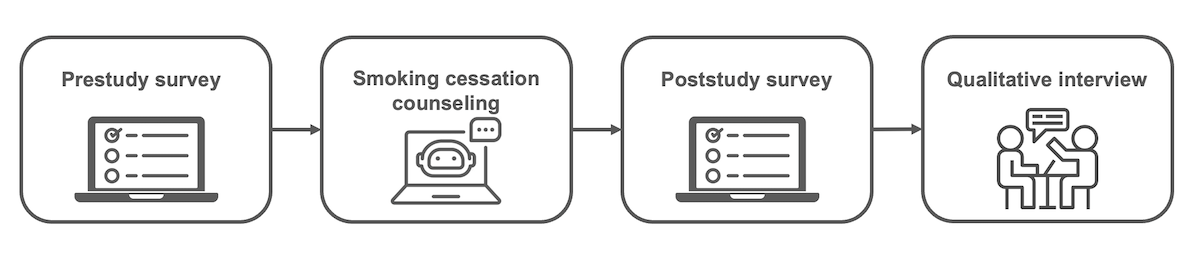
Mixed methods study design. Participants completed a pre-study survey, a single smoking-cessation counseling session with Aipaca, a post-study survey, and an optional qualitative interview. Quantitative pre-post outcomes (preparedness or readiness, self-efficacy, and knowledge) were triangulated with conversation-analysis of transcripts and thematic analysis of interviews to explain observed changes and user experience. Population: adult US smokers (≥5 cigarettes per day) recruited via MTurk.

We conducted an observational, single-arm, mixed methods study using methodological triangulation to develop a comprehensive understanding of both outcomes and underlying mechanisms of GenAI-enabled smoking cessation counseling. The study design integrated three complementary components: (1) quantitative pre-post surveys to assess changes in preparedness for cessation, (2) conversation analysis of counseling transcripts to examine communication dynamics during human-chatbot interaction, and (3) qualitative thematic analysis of semistructured interviews to explore user perceptions and identify design implications (Figure 3). This triangulated approach is well suited for evaluating complex communication interventions, as it aligns proximal behavioral outcomes with observed interaction processes and user meaning-making [12].

### Ethical Considerations

This study was reviewed and approved by the institutional review board (IRB) of the University of Oklahoma (IRB15693). All procedures complied with institutional policies and applicable regulations for research involving human participants. Prior to any study activities, participants reviewed an IRB-approved information sheet and provided electronic informed consent within a secure survey environment. Participation was voluntary, and respondents could withdraw at any time without penalty. To protect privacy, enrollment and follow-up occurred through MTurk (Amazon Mechanical Turk), single participation was enforced via Worker ID with IP/device checks, and all data were stored on access-controlled servers. Monetary compensation was provided through MTurk after verification of task completion. Before participant enrollment, we evaluated Aipaca’s performance with a standard TTS certification examination required by the Council for Tobacco Treatment Training Programs–accredited courses; Aipaca scored 81% (70% passing threshold). These preparatory evaluations were used to confirm content alignment with evidence-based counseling standards and to minimize participant risk prior to initiating data collection.

### Participants and Recruitment

Participants were recruited via MTurk between May and June 2024. Eligibility criteria included (1) currently smoking ≥5 cigarettes per day, (2) at least 18 years of age, (3) residing in the United States, and (4) having a ≥97% approval rate on human intelligence tasks on MTurk. Twenty-nine participants (16 males, 13 females; mean age 45.5, SD 12.5, range 25-65 years) completed the pre-post surveys and counseling session, of whom 25 completed the semistructured interview. Descriptive smoking history and nicotine dependence characteristics are summarized in Table S1 in Multimedia Appendix 1, with Fagerström Test for Nicotine Dependence (FTND) categories reported to contextualize likely withdrawal severity and inform the therapeutic tailoring recommended in clinical practice.

Recruitment used systematic quality and uniqueness safeguards. We limited enrollment to one submission per Worker ID, implemented IP/device duplication checks, embedded attention-check items in both screening and baseline instruments, and prevented back-button resubmissions. Geo-IP verification confirmed US access. MTurk completion codes were generated only upon successful postsurvey submission and were validated against Worker IDs prior to payment to prevent duplicate participation. Survey data were transmitted over Transport Layer Security and stored on a secure server; the linkage file between Worker ID and survey ID was maintained separately and deleted after payments were issued.

### Procedures

#### Pre-Post Survey Measures

After consent, participants completed a preintervention survey on Qualtrics measuring demographics, tobacco use (years of smoking, cigarettes per day, prior quit attempts, and longest abstinence), nicotine dependence (FTND) [33], and cessation preparedness. Preparedness included (a) perceived sufficiency of knowledge about smoking-related health risks (0-100) and cessation methods (0-100) [34,35], (b) cessation self-efficacy (1-7) [36], and (c) readiness to quit (10-point readiness ladder) [37]. The same preparedness measures were collected again following the counseling session.

#### Counseling Session With Aipaca

Participants then engaged in a text-based initial counseling session with Aipaca, a GenAI chatbot designed to deliver evidence-informed tobacco treatment (eg, brief advice, assessment, and collaborative planning) and MI microskills (open questions, reflections, affirmations, and summaries). The chatbot was implemented via GPT-4 guided by a system-level prompt specifying counseling goals, turn-taking structure, safety guardrails (eg, escalation for medical danger and avoidance of diagnostic claims), and content boundaries. A programmatic scaffold ensured coverage of core counseling elements (history, motivations, barriers/triggers, quit-aid education, and collaborative plan) while allowing free-text responses.

#### Semistructured Interviews

Following the postsession survey, participants were invited to a 30-minute semistructured interview exploring (1) perceived value and critiques of the chatbot session, (2) opportunities and challenges for real-world implementation, and (3) design suggestions regarding interaction style, features, and safeguards. Twenty-five interviews were completed, as four participants (participants 6, 10, 25, and 27) opted out after the postintervention survey due to time constraints.

#### Reporting Standards

We reported this mixed methods feasibility study in accordance with the GRAMMS (Good Reporting of a Mixed Methods Study) guideline [38-40].

### Data Sources and Analysis

#### Quantitative Analysis

We used paired-samples *t* tests to assess pre-post change in each preparedness measure. For interpretability, we report mean differences with 95% CIs and standardized within-person effect sizes (Cohen *d_z_*). Analyses were conducted with 29 participants.

#### Conversation Analysis of Counseling Transcripts

We analyzed 29 counseling transcripts (1514 total turns; session range=50-112 turns) using a microsequential approach to characterize how counseling interactions unfolded turn by turn (eg, adjacency pairs, question-answer sequences, formulations, and contextual referencing of prior talk) and how these sequences supported personalization and collaborative plan formation. Two authors (YL and RS) met weekly to iteratively develop and refine an interactional codebook that included categories such as topic initiation and uptake, referencing earlier content, agenda-setting, formulation and summarization, and plan negotiation. Discrepancies were resolved through discussion until consensus was achieved.

#### Thematic Analysis of Interviews

Interview data were transcribed and analyzed using reflexive thematic analysis focused on three a priori domains: (1) perceived values and criticisms, (2) opportunities and challenges of GenAI chatbot for counseling support, and (3) design feedback. Initial codes were generated by the corresponding author (RS) following established procedures [41], and two additional authors (YL and PC) independently reviewed a subset to refine code definitions and theme boundaries. Disagreements were resolved by consensus. The final thematic structure included 3 overarching themes and 11 subthemes (Table S2 in Multimedia Appendix 1), with exemplar participant quotes selected to illustrate each theme.

## Results

### Pre-Post Changes in Preparedness for Cessation

**Figure 4:**
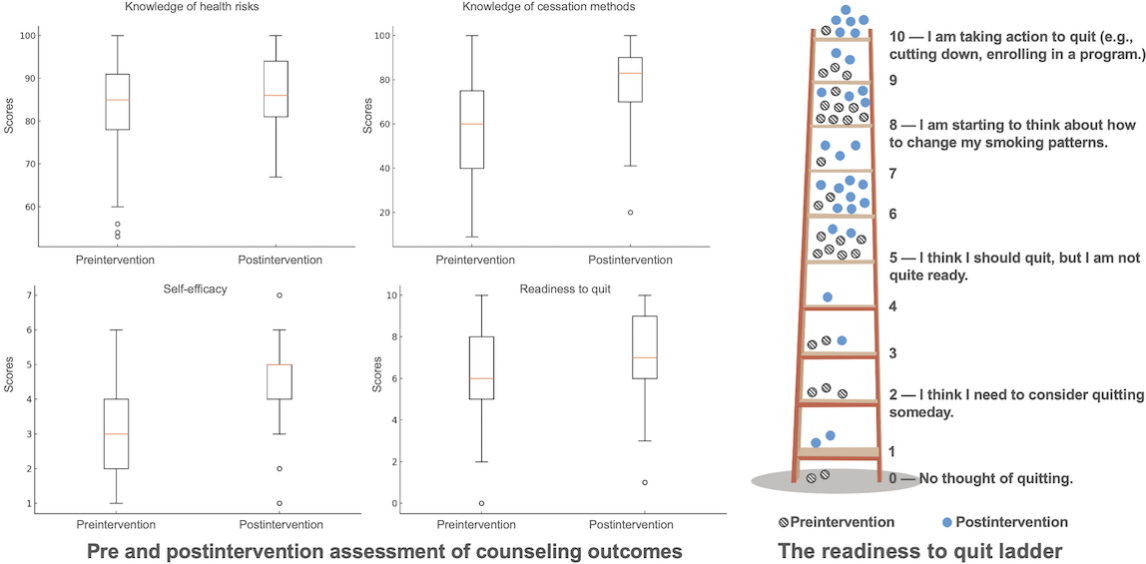
Pre-post changes in preparedness for cessation. The left panel displays four boxplots comparing pre- and postintervention scores for knowledge of health risks (0-100), knowledge of cessation methods (0-100), self-efficacy (1-7), and readiness to quit (0-10). The right panel illustrates the readiness to quit ladder, with shaded circles representing preintervention data and blue circles representing postintervention data. Within-person pre-post comparisons were conducted (2-sided α=.05).

Participants demonstrated statistically significant improvements across all 4 cessation preparedness indicators (Figure 4). On the 0-100 scales, perceived knowledge about smoking-related health risks increased from 82.59 (SD 13.35) to 86.21 (SD 8.69), t_28_=−2.12, *P*=.043, 95% CI 0.13-7.11, and *d_z_*=0.39. The *t* tests conducted in this study were 2-tailed. Knowledge of cessation methods increased from 56.86 (SD 24.02) to 76.86 (SD 18.50), t_28_=−6.77, *P*<.001, 95% CI 13.95-26.05, and *d_z_*=1.26. On the 1-7 scale, cessation self-efficacy improved from 3.10 (SD 1.32) to 4.38 (SD 1.52), t_28_=−5.05, *P*<.001, 95% CI 0.76-1.79, and *d_z_*=0.94. On the 10-point readiness ladder, readiness to quit increased from 5.76 (SD 2.80) to 6.83 (SD 2.52), t_28_=−4.08, *P*<.001, 95% CI 0.53-1.61, and *d_z_*=0.76. Of the 29 participants, 19 (65.5%) moved up at least one rung on the readiness ladder, more often shifting from contemplation (eg, “I should quit, but I’m not quite ready”) to preparation (eg, “I am starting to think about how to change my smoking patterns”) or initial action (eg, “I am taking actions to quit”).

### Communication Dynamics During Counseling Sessions

The microsequential analysis of 29 transcripts offered insights into the interactional dynamics of human-chatbot counseling sessions, illuminating not only *what* transpired but also *how* the counseling work was co-constructed turn by turn [42]. Three recurrent patterns were identified: contextual referencing and continuity, formulations with elaboration prompts, and narrative progression toward collaborative planning.

#### Contextual Referencing and Continuity

Both participants and the chatbot regularly referenced prior talk (eg, earlier-stated reasons for quitting, identified triggers, or previous quit attempts) to maintain topical continuity and conversational coherence. For instance, when Participant 13 revisited a prior cardiac hospitalization as a motivation for quitting, the chatbot reformulated and extended this into a concrete health benefit:

Participant 13: Like I said before, the main reason I need to quit is my heart. I really want to stay healthy and live a long life.

Aipaca: That is a compelling reason to quit. You know, quitting can really make a huge difference for your heart. It cuts the risk of heart disease and can help your body recover over time. You might even notice better stamina as you get further along in the process.

#### Formulations, Clarifications, and Elaboration Prompts

The chatbot frequently restated participants’ prior input and followed with clarifying or elaborative questions (eg, “You mentioned stress as an important trigger, can you tell me more about which situations set it off?”), encouraging deeper disclosure around triggers and coping contexts [43]. For example, Participant 23 mentioned stress-related cravings, and the chatbot prompted further elaboration:

Participant 23: I tend to get cravings when I’m stressed.

Aipaca: I see, stress is a big one for a lot of people. Can you tell me a bit more about what kinds of stress tend to trigger those cravings? Is it work, family, or maybe certain situations that really set it off?

#### Narrative Progression and Collaborative Planning

Sessions often followed a trajectory from orientation and history-taking into reflective exploration of motivations and barriers, culminating in collaborative plan building (eg, selecting a quit date, discussing quit aids, and drafting coping strategies). Early turns frequently used inclusive pronouns (eg, “we” and “let’s”) to facilitate affiliation and joint problem-solving, as in “Let’s dig into it” and “Now we know better about what triggers you to smoke” [43]. As therapeutic rapport developed, the chatbot transitioned to more in-depth questions about previous quit attempts, strategies used, and the struggles encountered. The progression encouraged participants to move from brief factual responses to more personal disclosure of feelings, motivations, and barriers to quitting. For example, Participant 21 initially gave brief responses about smoking history (eg, “35 years”) but later shared detailed reflections on difficulties with cravings, stress, and sleep disruption during a past quit attempt:

The longest I managed to stay smoke-free was about a month. I was really trying, like sticking to it every day, but it was hard. The cravings were really intense, especially when I was stressed. I was feeling pretty irritable and had trouble sleeping too. But eventually, when things got tough, like work stress and some family stuff, I just caved and went back to smoking. It felt like the only way to deal with everything at the time.

This progressive nature of the counseling discourse allowed the chatbot to adapt to participants’ needs and preferences, fostering more personalized and supportive interactions. In later sequences, Aipaca pivoted toward readiness assessment and co-construction of tailored quit plans by linking motivations to actionable strategies:

Aipaca: You’ve mentioned family as a big reason for quitting. How do you think quitting will impact your relationship with them?

Participant 3: I think it’ll be good. I don’t want my kids to see me smoke, and I don’t want to be out of breath when I’m playing with them.

Aipaca: That sounds like a strong motivator. How do you feel about involving your family in your quit plan? Maybe they could support you when cravings hit or during stressful moments.

Taken together, these sequential practices, including contextual referencing, formulation with elaboration, and a progressive trajectory from orientation to collaborative planning, facilitated contingent, personalized counseling within sessions. The patterns suggest that the chatbot’s turn design consistently leveraged participants’ prior contributions to sustain coherence and scaffold the movement from information gathering toward actionable next steps aligned with their motivations and barriers.

### User Perceptions of Smoking Cessation Counseling With GenAI Chatbot

Most participants (n=18) indicated an intention to continue using Aipaca as a resource to support smoking cessation. Commonly cited strengths included accessibility, nonjudgmental nature, perceived empathy, and personalized responses. Several participants described Aipaca as a useful complement rather than a stand-alone solution:

A good starting point...but I would use it in conjunction with sessions with a specialist.

Participant 9

I would trust a doctor’s advice over a chatbot. But if I wanted some quick information, I would use it.

Participant 15

In contrast, 5 participants expressed reluctance to engage further with Aipaca, citing an impersonal tone, lack of accountability, or a communication style they felt was overly goal-directed. Notably, 8 participants reported no dislikes, underscoring the system’s overall acceptability.

### Perceived Value of Aipaca in Quit-Smoking

#### Reliable and Accessible Support Resource

Participants recognized Aipaca as a valuable source of accessible, always available, and high-quality cessation support. They emphasized the clarity, relevance, and simplicity of the information provided, describing responses as “very informative and detailed” [Participant 2], “fast and accurate” [Participant 24], “straightforward and clear...relevant to my initial inputs” [Participant 1], and “done so very well, wasn’t overly complex and seemed helpful” [Participant 17]. Several participants highlighted its potential as a comprehensive informational resource: “Aipaca gives me all the relevant answers I need without searching” [Participant 24].

Availability in moments of temptation was especially valued. Participants noted the benefit of having real-time support when cravings struck:

You can message all day when you have a craving, and it provides you motivation not to smoke.

Participant 12

Others emphasized its reliability as a stand-in for social support when traditional resources were unavailable:

Just having something there for support would be a great guide to quitting. A lot of times there may not be anyone around to talk to. But with Aipaca, you wouldn’t have to worry about that.

Participant 29

For participants with limited support networks, Aipaca was viewed as a valuable alternative: “valuable in cases where a person does not have a social support system in any other way” [Participant 3]. Some even preferred it to traditional supports, citing its less personal nature as advantageous:

As an introvert, I personally prefer the chatbot since it’s less personal than talking to a doctor.

Participant 26

#### Nonjudgmental and Motivating Interaction

Participants valued Aipaca’s humanlike and supportive qualities, describing the interaction as personal, encouraging, and motivating. As Participant 1 stated:

I would use Aipaca for the personality of the responses that it gave...as it resonated easily with me and would influence me to continue to use it in my quitting phase.

Participants frequently described the chatbot as “encouraging and supportive” [Participant 20], with several noting that it boosted their confidence and self-efficacy: “something that can constantly boost your mentality and help you feel important enough to quit” [Participant 12].

Several participants reported experiencing a sense of sincerity and care during interactions.

I felt heard and encouraged. I felt I could ask anything. The chatbot really had my best interest in mind.

Participant 23

Others echoed this:

It felt like I was talking with a real person, one who was concerned about me and wanted to help.

Participant 28

Importantly, Aipaca also created a safe, nonjudgmental space for discussing struggles. As Participant 5 noted:

I feel more comfortable chatting to it than talking to someone in my family who has never smoked and doesn’t understand how addicting it is.

#### Contingent, Personalized, and Interactive Communication

Participants perceived Aipaca as capable of understanding their inputs and generating relevant, contextually appropriate responses, laying the foundation for meaningful contingent communication.

I felt like Aipaca really understood what I was saying. There was no confusion or having to word my questions differently like when I have used other chatbots in the past...I felt heard and understood.

Participant 11

Similarly, Participant 14 emphasized its ability to maintain coherent, long-form dialogue:

It definitely understood what I said every single input. There was no confusing interaction, which even now is hard to do with a chat in a conversation that long.

Participants also highlighted its corrective capacity, as Participant 19 observed:

There were also a few times that I had misspelled a word, and it gave me a response that was related to the correct word. For example, I put “wait” and meant “weight,” but it gave me an answer for “weight.”

The chatbot’s ability to reference prior exchanges enhanced personalization and coherence:

It recalled the things I could do to replace the moments when I’m triggered, such as playing piano. When it seemed to recall and respond properly to what I was saying and was detailed in its response, it made me feel like I could really be helped.

Participant 7

Participants emphasized that Aipaca “seemed to listen to me...It didn’t seem like it just gave canned responses” [Participant 9], and several remarked on the suitability of its recommendations: “it answered questions that I had, and made recommendations suited to me specifically” [Participant 17].

Finally, participants valued the chatbot’s interactive qualities and conversational pacing.

It was very conversational; it understood my context and went “back and forth” very well.

Participant 8

Others appreciated features that mimicked human typing: “responses came back gradually instead of as one big chunk of text. It gave the illusion that the chatbot was typing in real-time” [Participant 3]. These qualities reinforced the perception of natural, 2-way dialogue.

### User Criticisms of Aipaca for Quit-Smoking

While participants generally emphasized Aipaca’s strengths, criticisms varied, reflecting individual differences in counseling needs and preferences. Notably, 8 participants reported no dislikes. Some participants felt Aipaca’s focus on smoking cessation left little room for personal connection or small talk, making the interaction feel “cold and clinical” [Participant 3] or “like a helicopter mom” [Participant 14]. Others found its strict goal orientation tiring:

The chatbot returned everything back to the topic at hand, which was tiring.

Participant 28

Yet preferences diverged; while some described the counseling style as pushy, others wanted more firmness:

It could be a little more compelling and stricter. I need a kick in the ass.

Participant 26

In addition, several participants noted that the impersonal nature of a chatbot reduced accountability:

I would be able to “cheat” and not be accountable...knowing it is only a computer.

Participant 7

Others pointed to limited cultural resonance:

I doubt it ever saw Pulp Fiction and saw how cool John Travolta looked smoking.

Participant 19

Aipaca’s response style also drew mixed reactions. Quick replies sometimes seemed insincere:

It answered within seconds...not seeming to have time to actually read what I wrote.

Participant 7

Conversely, a few found the delay between messages frustrating:

The seconds of response time felt very long...I thought I lost my internet.

Participant 26

### Opportunities and Challenges of GenAI Chatbots for Smoking Cessation

#### Enhance Understanding and Aid Decision-Making

Participants consistently highlighted Aipaca’s potential to expand knowledge about smoking’s effects and support informed decision-making. They valued access to accurate, comprehensive, and up-to-date information, including actionable guidance on cessation methods and medications:

It could help someone become knowledgeable on the effects of smoking and help them make decisions.

Participant 2

It could bring opportunities to provide the latest info on how to best quit smoking.

Participant 16

Some appreciated its ability to answer questions difficult to research independently:

I could ask Aipaca questions that I don’t know how to google.

Participant 8

However, concerns were raised about accuracy and reliability:

My only concern is it giving false or inaccurate information.

Participant 2

The chatbot has to be very accurate and not make mistakes.

Participant 4

#### Eliciting and Sustaining Motivation

Participants valued Aipaca’s capacity to provide continuous, on-demand encouragement and reminders to reinforce motivation:

It would be helpful in that it is always available on demand.

Participant 9

It could help remind me why I need to quit and offer support.

Participant 5

Several emphasized the importance of proactive engagement, such as follow-ups when inactive:

I would like it to check in with me if I haven’t used it in a while.

Participant 17

At the same time, barriers to sustained use were noted, including fading commitment, forgetting to engage during cravings, and technical issues:

I could see potential challenges such as users abandoning their goal completely and forgetting about the chatbot.

Participant 1

What if Aipaca was down for maintenance and I couldn’t get advice?

Participant 11

#### Long-Term Support and Companionship

Many participants viewed Aipaca as a potential companion and accountability partner:

I view it as a “friend” to stick with me even if I fail.

Participant 26

If used on a consistent basis, it would become my partner, and I wouldn’t feel alone.

Participant 28

Others saw broader applications for behavior change, such as dieting, or even societal benefits through reduced smoking prevalence:

I expect Aipaca to positively affect society and significantly lower the number of smokers.

Participant 20

Yet limitations of nonhuman support were evident. Some doubted its capacity for empathy: “It cannot truly understand what this process might be like psychologically” [Participant 9], while others questioned its accountability: “With a chatbot, there’s zero social pressure or consequences if I don’t follow through” [Participant 14].

### Design Feedback and Suggestions From Participants

Participants recommended design improvements to optimize Aipaca for long-term cessation support, emphasizing sustained engagement and stronger interpersonal connections.

#### Personalized, Gamified, and Proactive Engagement

Many participants suggested personalized reminders, adaptive progress tracking, and milestone recognition to reinforce motivation:

I would like it to keep track of my quitting, and to message me if I haven’t spoken to it lately.

Participant 17

A progress bar...with periodic reminders and check-ins.

Participant 28

Others wanted milestones tied to health or financial benefits, such as heart recovery or money saved [Participants 9 and 17]. Gamification was proposed to boost engagement, including points, badges, or rewards:

I could earn points for staying abstinent...making it fun would help me stay committed.

Participant 13

Points I could redeem for items...would be more motivating.

Participant 15

Participants also highlighted the value of proactive accountability, with some preferring motivational nudges and others requesting firmer reminders:

Keep me on task...chide me if I mess up.

Participant 28

It is objective, so I can be accountable without feeling guilty.

Participant 7

#### Anthropomorphic Design and Empathy

Participants emphasized the need for more humanlike and empathetic interactions. Suggested features included voice-based communication, visual aids, and avatars to make conversations feel natural and engaging:

Voice-to-text will make the experience much more convenient.

Participant 1

Charts or supportive images...would make it feel less like a wall of text.

Participant 3

Customizable personalities and tones were also requested:

Different people need different tones.

Participant 2

I want to choose a personality that matches mine.

Participant 23

Above all, participants stressed empathy and relatability as critical for emotional support:

Aipaca should reassure the user that their feelings are normal and encourage them to stay strong.

Participant 20

The most important feature is empathy...like someone in a support group who has also quit smoking.

Participant 3

Several suggested giving Aipaca the identity of a former smoker to strengthen credibility and connection:

It would be beneficial for the chatbot to have an identity as another smoker, sharing inspirational stories on how it quit successfully.

Participant 20

## Discussion

### Principal Findings

This study evaluated the feasibility of delivering evidence-based smoking cessation counseling through a GenAI chatbot using a triangulated mixed methods design. By integrating quantitative pre-post outcomes, qualitative interviews, and conversation analysis, we provide a multilayered account of how generative AI may support both the effectiveness and the relational dynamics of digital cessation counseling. In addition, we examined the communicative processes through which intervention effectiveness was achieved and situated user perceptions within broader theoretical debates in human-computer interaction and digital health.

### Quantitative Outcomes: Feasibility in the Context of Evolving Standards

Pre-post analyses revealed significant improvements in participants’ preparedness to quit smoking across knowledge, self-efficacy, and readiness indicators. These proximal effects extend meta-analytic findings that CAs can improve cessation outcomes by demonstrating that GenAI can achieve measurable motivational gains even in the initial counseling session [13,14]. While prior trials often tested scripted or retrieval-based systems with modest effect sizes, our findings suggest that generative models may accelerate short-term change by sustaining more naturalistic engagement. At the same time, our results must be interpreted cautiously. Considering that high attrition and methodological heterogeneity have hampered generalizability in prior CA studies [14], single-session effects risk inflating positive impressions due to novelty, and sustained engagement remains an open question. Moreover, as participants explicitly compared Aipaca to commercial LLMs such as ChatGPT, future trials must account for a shifting sociotechnical baseline: users now expect counseling systems to match the coherence and empathy of widely available generative models. This creates a higher threshold for both effectiveness and acceptability than in earlier CA research.

### Qualitative Insights: User Perceptions, Empathy, and Expectations

Interviews highlighted Aipaca’s perceived value as an accessible, nonjudgmental, and motivating resource, echoing earlier findings that empathy and personalization drive acceptability in digital cessation tools [18,44]. Importantly, participants did not report uncanny valley effects or any psychological reactance, which are commonly documented when chatbots simulate but fall short of human empathy [45,46]. Instead, they embraced Aipaca’s empathetic tone and requested more anthropomorphic features, such as distinct personalities, cultural resonance, and long-term relational continuity. This divergence suggests that uncanny valley discomfort may emerge primarily when a chatbot’s language capabilities misalign with its social role. By contrast, Aipaca’s generative fluency supported alignment between its assigned role (counselor) and user expectations, mitigating reactance. Yet critiques about accountability, cultural understanding, and rigid goal orientation highlight persistent gaps. Participants expressed concern that an impersonal chatbot could not provide social pressure to maintain abstinence or reflect insider knowledge of smoking culture. These limitations underscore Rogers’ client-centered counseling principle that therapeutic alliance depends not only on empathy but also on authenticity and shared understanding [47]. Users’ suggestions for gamified progress tracking, proactive reminders, and empathetic personas extend prior human-centered design research (eg, QuitBot and Florence), reinforcing the need to embed co-design processes in future development [22,25].

### Conversational Analysis: Contingency, Sequential Structure, and Therapeutic Work

The microsequential analysis of transcripts illuminated how generative AI supports counseling processes at the turn-by-turn level. Three recurrent practices—contextual referencing, formulation-plus-elaboration, and progression toward collaborative planning—demonstrated contingency, the property of conversations in which each utterance builds on prior talk [48]. This interdependence is critical for trust, perceived competence, and social connection [49,50]. By maintaining topical continuity, referencing earlier reasons for quitting, and scaffolding from orientation to collaborative plan-building, Aipaca enacted the very sequencing associated with MI and client-centered therapy. This extends the literature in two ways. First, it provides empirical evidence of process-level mechanisms rarely described in chatbot research. Prior reviews have highlighted relational communication as a gap in CA design [13], but few have shown how GenAI achieves such dynamics in practice. Second, it underscores the limitations of rigid scripting: while Aipaca could sustain coherent therapeutic narratives, its strict redirection of off-topic talk sometimes strained rapport. Future systems may need adaptive topic-switching, reinforcement learning, or sentiment-aware discourse modeling to balance counseling focus with the social nuances that participants valued.

### Implications for Practice and Policy

Generative AI chatbots could augment established services such as quitlines by providing continuous, low-intensity support between counselor calls. Embedding tools such as Aipaca into multilevel cessation infrastructures could increase reach and continuity, particularly for those with limited access to in-person treatment. However, evaluations of ChatGPT and related models show inconsistent adherence to cessation guidelines and occasional misinformation [51]. Clinical deployment, therefore, requires transparent validation protocols, integration of guideline-concordant knowledge bases, and escalation pathways to human specialists. Frameworks such as FUTURE-AI emphasize fairness, robustness, explainability, and traceability as essential for trustworthy health AI. Although chatbots may reduce access barriers for populations with limited social support, mobility, or geographic access to cessation services [18], their design must incorporate cultural adaptation, language inclusivity, and sensitivity to avoid reproducing inequities. Training on diverse linguistic and cultural datasets, together with participatory co-design involving underrepresented populations, will be essential to ensuring fair access and cultural relevance. Such inclusive design practices can strengthen trust, usability, and engagement across high-need populations and ensure that AI-augmented cessation tools contribute to equitable improvements in public health outcomes.

### Limitations and Future Work

This study has several limitations that inform directions for future research. First, its cross-sectional design restricts our ability to assess long-term cessation outcomes. While participants showed immediate improvements in preparedness indicators, it remains unclear whether these gains can be sustained over time. Future research should use longitudinal designs to evaluate Aipaca’s capacity to support maintenance, relapse prevention, and multisession counseling. Second, the relatively small sample size (n=29) limits statistical power and generalizability. Future studies with larger, more diverse samples would increase the ability to detect meaningful subgroup differences, such as baseline readiness to engage in a quit attempt or variability in digital literacy. Third, the absence of a control group precludes comparison with standard evidence-based approaches to tobacco cessation counseling. Future research should include randomized or noninferiority designs to evaluate Aipaca’s effectiveness relative to human-delivered counseling and existing digital interventions. Fourth, the within-subject, pre-post design precludes direct comparison with alternative chatbot architectures. Incorporating between-subject comparisons with rule-based or retrieval-based systems would clarify whether generative AI’s adaptive, personalized responses confer a distinctive advantage, both in counseling effectiveness and communication dynamics. Such comparisons could also illuminate how different system designs align with specific counseling goals. Fifth, participants varied in their stage of change, from contemplation to preparation and action. This heterogeneity may have influenced the perceived relevance of the single-session intervention, which emphasized readiness assessment, health education, and quit planning. Future studies should tailor chatbot content to the readiness stage, delivering more targeted, stage-appropriate support. Finally, the sample was predominantly White, limiting insight into the sociocultural factors shaping cessation experiences in minoritized groups. Smoking behavior and treatment engagement are deeply influenced by cultural norms, identity, and social context. Future research should prioritize the recruitment of more diverse populations to ensure that generative AI chatbots can be culturally responsive and equitably address the needs of high-need and underrepresented groups.

### Conclusions

This study reported findings from a mixed methods feasibility evaluation of Aipaca, a GenAI chatbot designed to deliver smoking cessation counseling. By integrating quantitative pre-post surveys, qualitative interviews, and conversation analysis, we demonstrated that GenAI can facilitate measurable improvements in cessation preparedness, while also enacting counseling-relevant communication dynamics such as contextual referencing, collaborative planning, and narrative progression. Our findings highlight both the promise and the challenges of deploying GenAI in behavioral health. Participants valued Aipaca’s accessibility, personalization, and empathy, yet also articulated expectations for richer social roles, cultural resonance, and long-term accountability. Design feedback emphasized the importance of personalized and proactive engagement, gamified progress tracking, and anthropomorphic features to sustain motivation and strengthen interpersonal connections. Viewed through a communication contingency lens, this study illustrates how GenAI reshapes the dynamics of human-chatbot interaction and redefines users’ expectations of digital health tools. More broadly, it offers practical guidance for implementing evidence-based interventions with GenAI chatbots: pair adaptive language generation with human-centered design, embed safeguards for accuracy and trust, and integrate systems into existing cessation infrastructures to ensure clinical relevance and equity.

## Appendix

Multimedia Appendix 1 Participants smoking history and nicotine dependence characteristics and the thematic analysis.

Multimedia Appendix 2 Full survey instruments.

Multimedia Appendix 3 Semistructured interview guide.

Multimedia Appendix 4 System prompt.

Multimedia Appendix 5 GRAMMS checklist.

## Abbreviations

ACT: acceptance and commitment therapy
AI: artificial intelligence
CA: conversational agent
CBT: cognitive behavioral therapy
FTND: Fagerström Test for Nicotine Dependence
GenAI: generative artificial intelligence
GRAMMS: Good Reporting of a Mixed Methods Study
IRB: institutional review board
LLM: large language model
MI: motivational interviewing
NLP: natural language processing
RCT: randomized controlled trial
RR: risk ratio
TTS: Tobacco Treatment Specialist

## Data Availability

Due to institutional review board (IRB) restrictions (IRB15693), raw counseling transcripts cannot be publicly shared because they may contain health-related information and potentially identifiable details. Deidentified excerpts are included in the manuscript. Deidentified summary data supporting the findings (anonymized pre-post survey data with codebook and aggregate counts for qualitative themes) and analysis scripts are available from the corresponding author upon reasonable request for noncommercial research, subject to an IRB-consistent data use agreement. The full survey instruments and interview protocol are provided in Multimedia Appendices 2 and 3, and a partial system prompt is provided in Multimedia Appendix 4. Materials subject to third-party licensing (eg, Tobacco Treatment Specialist curriculum content embedded in prompts) cannot be redistributed.
