# Supplementary material for: AI-Enabled Personalized Smoking Cessation Intervention With the Aipaca Chatbot: Mixed Methods Feasibility Study
Source: JMIR Form Res. 2025 Dec 11;9:e73319. doi: 10.2196/73319 (PMC12741657; doi:10.2196/73319)
Supplement: Multimedia Appendix 1 [file formative_v9i1e73319_app1.docx]

**Table S1.** Participants demographics, tobacco use, and nicotine dependence level.

| **Participant** | **Age** | **Sex** | **Years of smoking** | **Cigarette/day** | **Nicotine dependence** |
| --- | --- | --- | --- | --- | --- |
| P01 | 29 | Male | 7 | 6 | Low |
| P02 | 25 | Male | 7 | 20 | Low |
| P03 | 36 | Female | 10 | 8 | Low |
| P04 | 30 | Male | 10 | 6 | Low |
| P05 | 42 | Female | 10 | 8 | Low |
| P06 | 34 | Male | 13 | 20 | Low |
| P07 | 65 | Female | 15 | 8 | Low |
| P08 | 33 | Male | 17 | 18 | Low |
| P09 | 34 | Male | 17 | 11 | Low |
| P10 | 35 | Male | 17 | 20 | Low |
| P11 | 34 | Female | 18 | 5 | Low |
| P12 | 34 | Female | 18 | 10 | Very Low |
| P13 | 53 | Male | 20 | 16 | Low |
| P14 | 38 | Male | 21 | 15 | High |
| P15 | 39 | Female | 22 | 15 | Low |
| P16 | 40 | Male | 23 | 20 | Low |
| P17 | 44 | Female | 25 | 20 | Very Low |
| P18 | 42 | Male | 26 | 15 | Low |
| P19 | 42 | Male | 26 | 20 | Moderate |
| P20 | 52 | Female | 31 | 20 | Low |
| P21 | 63 | Male | 35 | 34 | High |
| P22 | 58 | Female | 38 | 15 | Low |
| P23 | 55 | Female | 40 | 20 | Moderate |
| P24 | 58 | Female | 41 | 15 | Low |
| P25 | 58 | Male | 42 | 30 | Low |
| P26 | 63 | Female | 43 | 20 | Low |
| P27 | 58 | Male | 45 | 15 | Low |
| P28 | 63 | Male | 45 | 8 | Low |
| P29 | 61 | Female | 45 | 20 | Low |

**Table S2.** Thematic analysis and exemplary quotations of user perceptions of Aipaca from interviews (N=29).

| **Domains** | **Subthemes** | **Exemplary quotations** |
| --- | --- | --- |
| Value and Criticism | Reliable and Accessible Support Resource | [The responses were] “informative and detailed” (P2), “fast and accurate” (P24), “straightforward and clear …relevant to my initial inputs” (P1), and “done so very well, wasn’t overly complex and seemed helpful” (P17). “Aipaca gives me all the relevant answers I need without searching” (P24).  “Having Aipaca would be helpful to give support when I feel like cheating and grabbing a smoke” (P22).  “You can message all day when you have a craving, and it provides you motivation not to smoke” (P12); “Just having something there for support would be a great guide to quitting. A lot of times there may not be anyone around to talk to. But with Aipaca, you wouldn’t have to worry about that” (P29); “It would give me help at any time I needed it. It would be able to give me real-time solutions to get through” (P23).  “Valuable in cases where a person does not have a social support system in any other way” (P3); “Easy way to get support and information, especially for a person without a supportive social group” (P20).  “As an introvert, I personally prefer the chatbot since it’s less personal than talking to a doctor” (P26); “I don’t like talking on the phone so I would not use a hotline for help. It is easy to chat to the bot and get advice and support” (P5). |
|  | Non-Judgmental and Motivating Interaction | “I would use Aipaca for the personality of the responses that it gave … as it resonated easily with me and would influence me to continue to use it in my quitting phase” (P1); “Very much like a counseling session with a human I would use” (P21); “Something that can constantly boost your mentality and help you feel important enough to quit” (P12); “It would also increase confidence even if it was just chatbot have something say you can do it” (P13).  “I felt heard and encouraged. I felt I could ask anything. The chatbot really had my best interest in mind” (P23); “very responsive…It seemed like an interested person” (P26); “It felt like I was talking with a real person, one who was concerned about me and wanted to help” (P28).  “I feel more comfortable chatting to it than talking to someone in my family who has never smoked and doesn’t understand how addicting it is” (P5); “I didn’t feel like a second-class citizen” (P7); “I feel like if I was going through a withdrawal, I could talk to it without judgment” (P23). |
|  | Contingent, Personalized, and Interactive Communication | [The responses were] “relevant to my initial inputs” (P1), “always interconnected to what we were talking about” (P14), and “aware of previous responses” (P16).  “I felt like Aipaca really understood what I was saying. There was no confusion or having to word my questions differently like when I have used other chatbots in the past… I felt heard and understood” (P11); “It definitely understood what I said every single input. There was no confusing interaction, which even now is hard to do with a chat in a conversation that long” (P14).  “There were also a few times that I had misspelled a word, and it gave me a response that was related to the correct word. For example, I put ‘wait’ and meant ‘weight,’ but it gave me an answer for ‘weight’” (P19).  “It recalled the things I could do to replace the moments when I’m triggered, such as playing piano. When it seemed to recall and respond properly to what I was saying and was detailed in its response, it made me feel like I could really be helped” (P7); “seemed to listen to me… It didn’t seem like it just gave canned responses” (P9); “it answered questions that I had, and made recommendations suited to me specifically” (P17); “I also liked when it asked me what I would do in various situations and then gave multiple suggestions and tips that work for me, instead of just instructing me” (P3).  “It was very conversational; it understood my context and went ‘back and forth’ very well” (P8); “responses came back gradually instead of as one big chunk of text. It gave the illusion that the chatbot was typing in real-time” (P3). |
|  | Unmet Social Expectations | “I didn’t like that there were no personal touches outside of talking about quitting smoking, no chit-chat or getting to know me as a person. It felt very cold and clinical” (P3); “like a helicopter mom” (P14); “It was very focused on the single goal of quitting smoking, which I suppose is appropriate. But when talking about such a difficult subject I find it easier to manage when I can pull away and distract myself for a minute every once in a while. The chatbot returned everything back to the topic at hand, which was tiring” (P28).  “It could be a little more compelling and stricter. I need a kick in the ass” (P26). |
|  | Lack of Accountability and Cultural Understanding | “It is too impersonal, and I would be able to ‘cheat’ and not be accountable for what I do regarding smoking…knowing it is only a computer talking to me and not a person, I would think I’d still be able to go on with my smoking habits and would eventually just stop using it” (P7).  “I also felt like the chatbot had a hard time relating to smokers. I doubt it ever saw Pulp Fiction and saw how cool John Travolta looked smoking” (P9). |
|  | Mechanistic Nature and Response Time | “It certainly seemed to answer within seconds, very quickly, not seeming to have time to actually read what I wrote. That made me feel not listened to” (P7).  “The seconds of response time felt very long…I thought I lost my internet” (P26). |
| Opportunities and Challenges | Enhance Understanding and Aid Decision-making | “It could help someone become knowledgeable on the effects of smoking and help them make decisions. I expect it to be accurate. I also expect follow-ups to everything” (P2). “It could offer new insight or knowledge. I could ask Aipaca questions that I don’t know how to google or need help understanding” (P8). “It could bring opportunities to provide the latest info on how to best quit smoking. I expect it to be up to date in this regard” (P16); “I expect good advice and clear actionable things to do (e.g., take medications)” (P21).  “My only concern is it giving false or inaccurate information” (P2); “The chatbot has to be very accurate and not make mistakes” (P4). |
|  | Eliciting and Sustaining Motivation | “It would be helpful in that it is always available on-demand. If someone needs help at a moment’s notice, a chatbot could be a realistic way to provide help” (P9). “It could help remind me why I need to quit and offer support.” (P5); “I would expect it to give me advice and recommendations for how to stay on track. I would also expect it to give me motivation.” (P17).  “I would also like it to check in with me if I haven’t used it in a while, to make sure I was staying on track and making progress” (P17).  “I could see potential challenges such as users abandoning their goal completely and forgetting about the chatbot” (P1); “Just remembering to use it if I’m having a bad day and want to smoke” (P5). “What if Aipaca was down for maintenance and I couldn’t get advice?” (P11); “I would be concerned about the availability of a chatbot… If I was having a bad craving and my internet was down, I’d probably succumb to having a cigarette” (P15). |
|  | Long-Term Support and Companionship | “I view it as a ‘friend.’ I expect it to stick with me even if I fail” (P26); “If used on a consistent basis, it would become my partner, and I wouldn’t feel alone in attacking this difficult challenge” (P28). “I think it would give me peace of mind that there would be something to talk to and go over my plan and keep me to it. It would let me know how successful I was” (P29).  “The opportunities it could bring are it could help me change my thinking also about dieting and weight, which is why I started smoking to some extent in the first place” (P7). “I expect Aipaca to positively affect society and significantly lower the number of smokers. I also think it would make a smoker’s quitting success rate higher than a human specialist” (P20).  “It cannot truly understand what this process might be like psychologically, as it cannot actually experience what smoking is like” (P9). “I know it is not a real person and only a computer. That would make me not ‘believe’ it and take its advice” (P7). “I like to say, if I make a commitment to quit smoking with my mom, wife, or a friend, I have to face the music if I go back on it. But with a chatbot, there’s zero social pressure or consequences if I don’t follow through” (P14). “I think the chatbot could not offer the same personalized support that a human could. There is a limit to the helpfulness of the chatbot based on the unique needs of various people” (P24). |
| User-Centered Design Feedback | Personalized, Gamified, and Proactive Approach for Sustaining Engagement | “I would like it to keep track of my quitting, and to message me if I haven’t spoken to it lately” (P17); “A progress bar, perhaps, with a projected date of being smoke-free, and periodic reminders and check-ins after that date to reinforce the progress made” (P28). “The bot could help me keep track of exact milestones to keep me motivated. It could tell me how much money I am saving to a fairly exact degree. It could maybe tell me what percentage of damage to myself I have undone” (P9); “I would like it to tell me things like, ‘You have quit smoking for one month now, so your heart rate is returning to normal,’ to acknowledge my milestone” (P17).  “I could earn points for staying abstinent for one day and level up as I accumulate points. The chatbot could award badges for achieving specific milestones and provide challenges like ‘stay smoke-free for 7 consecutive days.’ Making it fun would help me stay committed” (P13); “It could give me points or something for the days that I don’t smoke and then I could use those points to get items like a water bottle, exercise mat, coffee mug, etc. That would be more motivating” (P15).  “Keep me on task and don’t give me any excuses to bypass or duck it. I need it to support me, chide me, remind me, and don’t let me slack off. And if I do, it needs to help me back on the path” (P28); “I would want it to motivate me and encourage me and chide me if I mess up. I would want it to hold me accountable” (P17); “A chatbot would perhaps remind me several times throughout the day of the goals and specific benefits, dangers, and alternatives I could use. I can be accountable without worrying about ‘hurting’ its feelings if I don’t agree or if I get mad. It is objective, so that gives me freedom to not feel guilty” (P7). |
|  | Anthropomorphic Design for Enhancing Interpersonal Connections | “Features such as voice-to-text will make the experience much more convenient” (P1); “It would be nice if the chatbot could send images, like charts, or calendars, or even just supportive ‘you’ve got this’ image. This would make it feel more like a chat, and less like a wall of text” (P3).  “I think it needs to be kind and lifelike. A chatbot needs to feel human enough so that most people who use it think they are talking to someone real” (P4); “Customizing the chatbot answers to whoever it’s talking to. Different people need different tones” (P2); “I want to choose the chatbot with a personality that matches mine. It would make it more humanlike and caring” (P23).  “Aipaca should reassure the user that their feelings are normal and encourage them to stay strong” (P20); “It would be like having a best friend beside you, helping and pushing you to keep going” (P29).  “The chatbot needs to really understand the allure of smoking, not just the addiction. It has to grasp these complexities to help me quit” (P14); “The most important feature is empathy. The chatbot should feel like someone in a support group who has also quit smoking” (P3); “If Aipaca can’t be empathetic, then it can’t understand what motivates people to smoke, or to want to quit” (P8). “It would be beneficial for the chatbot to have an identity as another smoker, sharing inspirational stories on how it quit successfully. Including pictures would make it more relatable” (P20); “The chatbot should sound like someone who used to smoke and understood the appeal. I smoke not just because I’m addicted, and the chatbot needs to understand that before it can help” (P14). |
