# Supplementary material for: AI-Enabled Personalized Smoking Cessation Intervention With the Aipaca Chatbot: Mixed Methods Feasibility Study
Source: JMIR Form Res. 2025 Dec 11;9:e73319. doi: 10.2196/73319 (PMC12741657; doi:10.2196/73319)
Supplement: Multimedia Appendix 2 [file formative_v9i1e73319_app2.docx]

**Measurement Table**

**A.1 Pre-survey**

Socio-demographic factors

| **Measures** | **Scale** | **Variable** |
| --- | --- | --- |
| What is your age in years? |  | Age |
| What sex were you assigned at birth? | 1-Male  2-Female  3- Intersex  4- None of these | Sex |
| Are you Hispanic or Latino? | 1-Yes  2-No | Ethnicity |
| What is your race? (Select all that apply) | 1- White  2- Black or African American  3- American Indian or Alaska Native  4- Asian  5- Native Hawaiian or Pacific Islander  6- Other | Race |

Smoking behavior

| **Measures** | **Scale** | **Variable** |
| --- | --- | --- |
| How many years have you smoked cigarettes? (Fill in the number only) |  | smoking year |
| How many cigarettes do you usually smoke on an average day? (Fill in the number only) |  | cigarette per day |
| How many times in your whole life have you quit smoking for at least 24 hours? (If you have not attempted to quit, put 0) |  | quit attempt |
| Since you first started smoking, what was the longest period of time that you were able to stay off cigarettes? (In number of days)  (If you never quit, put 0. If your longest quit lasted more than 3 years, put 999). |  | Length of quitting |
| How often have you used each of the following in your efforts to quit smoking?  (1) Cold turkey (quitting all at once)  (2) Gradually cutting down  (3) Using medications like nicotine gum, patches, or champix  (4) Professional stop smoking clinic  (5) Hypnosis  (6) Support group  (7) Self-help manuals of books  (8) Other (please specify) | 1-Never  2-Seldom  3-Occasionally  4-Frequently  5-Repeatedly | Quitting strategy |
| This is a ladder where each rung on this ladder represents where various smokers are in their thinking about quitting smoking. Write down the number that indicates where you are now. 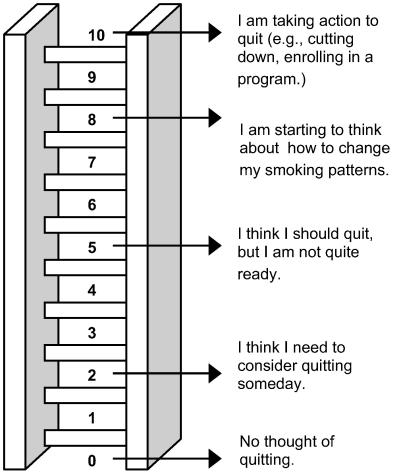  I am at rung ______ |  | readiness_before |
| How confident are you that you will be able to stop smoking at this time? | 1-Not at all confident  7-Extremely confident | Efficacy |
| Now, we would like you to rate your knowledge about health risks related to smoking. Please use a scale of 0 to 100, where 0 means knowing nothing and 100 means knowing everything you could possibly know about this topic. Using this scale, how much do you think you currently know about health risks related to smoking? | 0-100 (slider) | Knowledge Insufficiency |
| Now, we would like you to rate your knowledge about how to quit smoking. Please use a scale of 0 to 100, where 0 means knowing nothing and 100 means knowing everything you could possibly know about this topic. Using this scale, how much do you think you currently know about how to quit smoking? | 0-100 (slider) | Knowledge Insufficiency |

Nicotine dependence (Fagerstrom Test)

| **Measures** | **Scale** | **Variable** |
| --- | --- | --- |
| How soon after waking do you smoke your first cigarette? | 1- Within 5 minutes  2- 5-30 minutes  3- 31-60 minutes  4- After 60 minutes | FND_1 |
| Do you find it difficult to refrain from smoking in places where it is forbidden (e.g., in church, at the library, in the cinema)? | 1- No  2- Yes | FND_2 |
| Which cigarette would you hate most to give up? | 1- The first one in the morning  2- Any other | FND_3 |
| How many cigarettes per day do you smoke? | 1- 10 or less  2- 11 to 20  3- 21 to 30  4- 31 or more | FND_4 |
| Do you smoke more frequently during the first hours after waking than during the rest of the day? | 1- No  2- Yes | FND_5 |
| Do you smoke when you are so ill that you are in bed most of the day? | 1- No  2- Yes | FND_6 |

**A.2 Post-survey**

| **Measures** | **Scale** | **Variable** |
| --- | --- | --- |
| This is a ladder where each rung on this ladder represents where various smokers are in their thinking about quitting smoking. Write down the number that indicates where you are now. 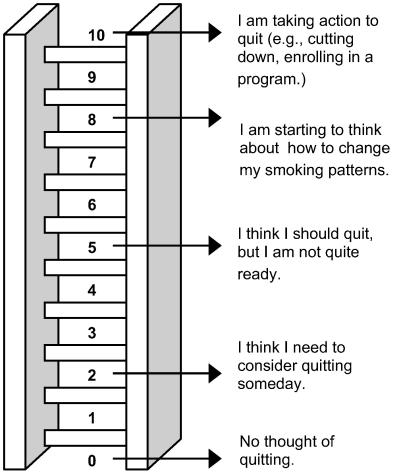  I am at rung ______ |  | Readiness_after |
| Now, after your conversation with the chatbot, we would like you to rate your knowledge about health risks related to smoking. Please use a scale of 0 to 100, where 0 means knowing nothing and 100 means knowing everything you could possibly know about this topic. Using this scale, how much do you think you currently know about health risks related to smoking? | 0-100 (slider) | Knowledge Insufficiency |
| Now, after your conversation with the chatbot, we would like you to rate your knowledge about how to quit smoking. Please use a scale of 0 to 100, where 0 means knowing nothing and 100 means knowing everything you could possibly know about this topic. Using this scale, how much do you think you currently know about how to quit smoking? | 0-100 (slider) | Knowledge Insufficiency |
| How confident are you that you will be able to stop smoking at this time? | 1-Not at all confident  7-Extremely confident | Efficacy |
