# Supplementary material for: AI-Enabled Personalized Smoking Cessation Intervention With the Aipaca Chatbot: Mixed Methods Feasibility Study
Source: JMIR Form Res. 2025 Dec 11;9:e73319. doi: 10.2196/73319 (PMC12741657; doi:10.2196/73319)
Supplement: Multimedia Appendix 3 [file formative_v9i1e73319_app3.docx]

**Semistructured Interview Guide**

0) Opening & Consent

- Confirm consent to participate and be recorded; explain confidentiality and voluntary participation. “Before we start, any questions about the study or today’s interview?”

1) Warm-up & Context

- “In a sentence or two, tell me about your current smoking and any recent thoughts about quitting.”
  - Probes: typical smoking day; prior quit attempts; supports used.

2) Walk-through of the Aipaca Session

- “Please walk me through your chat with Aipaca, from start to finish. What stood out?”
  - Probes: key moments; points of confusion; parts that felt helpful/not helpful; tone and pacing.

3) Perceived Value & Critiques

- “What did you like about the quit-smoking chatbot, and why?”
  - Probes: clarity/usefulness of information; empathy/support; personalization; timing/availability; naturalness of the conversation; references to your own inputs.
- “What did you not like, and why?”
  - Probes: tone (too pushy/too passive); repetition; going off-topic vs. redirecting too quickly; accountability; cultural resonance; trust/accuracy.
- “What do you think about using a chatbot for quitting smoking? Is it something you’d use?”
  - Probes: situations you would/wouldn’t use it; compare to human counselor, apps, or ChatGPT-style tools; single-session vs. ongoing use.
- “What would make you want to use a quit-smoking chatbot regularly?”
  - Probes: reminders/check-ins; empathy; convenience; evidence-based guidance; tracking progress or money saved; support during cravings.

4) Opportunities & Challenges for Real-World Use (6–8 min)

- “Thinking about using a chatbot to quit, what opportunities could it bring, and what would you expect from it?”
  - Probes: learning new strategies/medications; just-in-time support; accountability partner/companion; integration with quitlines or clinicians.
- “What challenges or concerns might come up?”
  - Probes: accuracy/misinformation; safety guardrails; privacy/data use; access (connectivity, literacy, disability); staying engaged; relapse handling; cultural fit.

5) Design Feedback: Features, Style, Safeguards (6–8 min)

- “When designing a chatbot to help people quit, which features are most important?”
  - Probes: quit-plan builder; trigger/craving coaching; medication education; stage-of-change tailoring; relapse recovery plans; referrals/escalation to humans.
- “If you were to use a chatbot to quit, what would you need it to do for you specifically?”
  - Probes: frequency of check-ins; personalization knobs (tone/persona); voice or visuals; language options; accessibility needs.
- “Do you have any suggestions to make the chatbot better?”
  - Probes: proactive nudges; gamification (points/badges/milestones like health gains or money saved); summaries after sessions; ‘coach’ vs ‘peer’ persona; identity (e.g., an ex-smoker story bank); transparency about limits and data.

6) Use Intention & Closing (2–3 min)

- “How likely are you to use Aipaca again?”
- “If you could change one thing about Aipaca right now, what would it be?”
- “Anything we didn’t cover that feels important?”
