# Supplementary material for: AI-Enabled Personalized Smoking Cessation Intervention With the Aipaca Chatbot: Mixed Methods Feasibility Study
Source: JMIR Form Res. 2025 Dec 11;9:e73319. doi: 10.2196/73319 (PMC12741657; doi:10.2196/73319)
Supplement: Multimedia Appendix 5 [file formative_v9i1e73319_app5.docx]

**GRAMMS checklist**

| **GRAMMS** | **Rationale** | **Page # in Manuscript** |
| --- | --- | --- |
| Rationale for using mixed methods. | To evaluate both outcomes (pre–post changes in preparedness, knowledge, self-efficacy) and mechanisms (how interactional features of the AI counseling session and user meaning-making relate to change). Mixed methods allow triangulation of surveys, conversation analysis, and interviews to generate meta-inferences about why changes occurred. | Methods: P5, P7. |
| Describe the design in terms of priority, sequence, and timing. | Convergent design with sequential timing: QUAN and QUAL strands were collected in one study visit (surveys immediately pre/post a single 10-15-min session; interviews scheduled post-session). Complementary priority (QUAN primary for outcomes; QUAL primary for explanation). Integration after separate strand analyses. | Methods: P7. |
| Describe each method (sampling, data collection, analysis). | QUAN: All consenting participants; pre-post surveys; paired tests with effect sizes and 95% CIs. QUAL-Conversation analysis: counseling transcripts coded for topic initiation and uptake, referencing earlier content, agenda-setting, formulation and summarization, and plan negotiation. QUAL-Interviews: Semi-structured interviews with completers (n=25); reflexive thematic analysis. | Methods: P7.  Interview guide, Appendix B. |
| Describe where/how integration occurred and who was involved. | Integration at analysis and interpretation via (1) joint displays aligning pre-post change with conversational features and interview themes; and (2) weaving in Results. Mixed team (communication scholars, Tobacco Treatment Specialists, HCI/AI researchers) conducted the integrative review. | Methods: P7.  Results: P8-12. |
| Limitations of integration. | Single-session exposure limits durability inference, and MTurk sampling may affect transferability | Limitation: P14. |
| Insights gained from mixing that could not be obtained by one method alone. | Mixed analysis linked specific conversational practices (e.g., contextual referencing) and user perceptions (usability/trust) to observed increases in preparedness and self-efficacy, which yields design implications (tailoring, guardrails) surveys alone would not reveal. | Methods: P7.  Results: P8-11.  Discussion: P13. |
